# A qualitative descriptive study to explore evaluation and effects of partnered rehabilitation research in Canada

**DOI:** 10.1186/s12961-026-01505-1

**Published:** 2026-06-26

**Authors:** Brenda J. Tittlemier, Juliette Cooper, Linda C. Li, Roberta L. Woodgate, Kathryn M. Sibley

**Affiliations:** 1 Rehabilitation Physiotherapy Department, Health Sciences Centre, RR140C-820 Sherbrook St., Winnipeg, MB R3A 1R9 Canada; 2 Department of Physical Therapy, College of Rehabilitation Sciences, Rady Faculty of Health Sciences, University of Manitoba, Winnipeg, MB Canada; 3 Department of Occupational Therapy, College of Rehabilitation Sciences, Rady Faculty of Health Sciences, University of Manitoba, Winnipeg, MB Canada; 4 Department of Physical Therapy, University of British Columbia, Vancouver, BC Canada; 5 Arthritis Research Canada, Vancouver, BC Canada; 6 College of Nursing, Rady Faculty of Health Sciences, University of Manitoba, Winnipeg, MB Canada; 7 Children’s Hospital Research Institute of Manitoba, Winnipeg, MB Canada; 8 College of Community and Global Health, Rady Faculty of Health Sciences, University of Manitoba, Winnipeg, MB Canada; 9 George & Fay Yee Centre for Healthcare Innovation, Rady Faculty of Health Sciences, University of Manitoba, Winnipeg, MB Canada

**Keywords:** Rehabilitation, Research partnerships, Assessment, Impact, Qualitative description

## Abstract

**Background:**

Partnered rehabilitation research, where researchers and knowledge users (e.g. clinicians, administrators, clients) collaborate on research decision-making and activities, may enhance the use of evidence in clinical practice. Previous research identified gaps in formal evaluation and demonstrated effects of the partnering process in rehabilitation research. This study explored evaluation and effects of partnering on the research process and outcomes, and how partnering contributed to those effects.

**Methods:**

We conducted a qualitative descriptive study. Eligible participants were researchers and knowledge users involved in partnered rehabilitation research in Canada who could understand English. We developed an interview guide on the basis of a published framework of partnering in research and a model of partnering in community-based research. We completed semi-structured interviews with seven researchers and six knowledge users, most of whom identified as women and White. Participants had a range of rehabilitation research experience from a few years to decades. We used reflexive thematic analysis to interpret the findings.

**Results:**

No participants formally evaluated their partnerships, citing challenges such as limited time, but believed it could be valuable. Accordingly, participants described many effects of partnering. We developed one main theme, “Partnering is an Interactive Process Shaping Research and those Involved,” along with three subthemes, “Partnering Influences the Research Process from Beginning to End,” “Learning through Partnering: Building Individual Research Knowledge, Skills and Capacity” and “The Effects of Partnering are Driven by Multiple Factors.” Participants described effects of partnering on the research process, outcomes and individuals; and factors that contributed to the effects.

**Conclusions:**

Despite the absence of evaluation, participants identified important impacts on research design, outcomes and individual learning that were influenced by an array of contributing factors. Persisting lack of evaluation of partnering in rehabilitation research may be restricting comprehensive understanding of the effects of partnering. Future research should prioritize approaches that balance rigor and relevance, and clarify who is best to lead evaluation activities.

**Supplementary information:**

The online version contains supplementary material available at https://doi.org/10.1186/s12961-026-01505-1.

## Background

Rehabilitation is a process consisting of the delivery of tailored, person-centred interventions for individuals with health conditions, which aim to enhance function, optimize quality of life, independence and social integration, minimize disability and maximize the ability to adapt to changes in an individual’s environment [1–4]. Rehabilitation should be evidence-informed to maximize health outcomes and improve the delivery of rehabilitation [5, 6]. However, there is a gap between rehabilitation evidence and uptake [7–10]. For instance, Moore et al. [9] conducted a survey which examined the evidence-based practices of rehabilitation clinicians working in southeastern Norway. Moore et al. [9] found that literature searching, critical appraisal and knowledge exchange were the predominant evidence-based activities, whereas the implementation of evidence to inform practice was least used. The existence of an evidence–practice gap is problematic because it may impact patient outcomes. For example, a pre-post observational study conducted by Linkewich et al. [11] examined the impact of an intervention to increase adoption of evidence-based practices in inpatient stroke rehabilitation. The authors found that enhancing adoption of evidence-based practice, i.e., minimizing the evidence–practice gap, resulted in improved motor function in patients [11]. Minimizing the evidence–practice gap is a complex process, which may explain its persistence [12, 13]. Several factors have been identified that influence the complexity of evidence use in practice including those specific to individuals (e.g., reluctance to change, expertise), and the organization (e.g., support from managers and colleagues to use evidence, a positive environment for evidence use and organizational policy) [12, 13].

While the complex process of minimizing the evidence–practice gap could partially explain why it persists, gaps between rehabilitation evidence and clinical practices may also stem in part from longstanding academic approaches in which researchers predominantly make decisions, without the involvement of intended users [14, 15]. Researcher-driven approaches develop evidence that, at times, has been perceived as irrelevant by users, thus resulting in the under-utilization of these findings [7]. As such, other approaches may be necessary to enhance rehabilitation evidence use in practice.

Some researchers are making efforts to enhance the uptake of evidence by partnering with knowledge users, i.e., any person, group or organization who can use the knowledge resulting from research to make informed healthcare decisions, during the research process [16–18]. These approaches may be referred to as research partnerships, an umbrella term that describes collaborative approaches to research [19–21]. The term “research partnership” describes the relationship between researchers and knowledge users [22]. Research partnerships may contribute to the enhanced uptake of findings because critical components of the research process address relevant priorities or incorporate knowledge user perspectives and experiences [23, 24]. Reported benefits of research partnerships include enhanced capacity, knowledge and skills of both researchers and knowledge users, improved community services, community empowerment and system changes by influencing policy [25].

In 2008, Wallerstein et al. [26] published the Community-Based Participatory Research (CBPR) model which could be used practically to help design, implement and evaluate partnered research. The CPBR model includes four domains: context (socio-economic characteristics, historical contributions, university and community capacities and health issue being studied); partnership dynamics (partnership structures, members and relationships); research/intervention and outcomes (systems, capacities, health and social justice) [26]. Belone et al. [27] subsequently validated the model and demonstrated how aspects of the partnering process, e.g., trust, affect intermediate and long-term effects [27]. In 2019, Jull et al. [20] conducted a systematic review that identified 54 frameworks of knowledge user engagement in health research. From the 54 frameworks, Jull et al. [20] reported 15 key concepts of engagement (Table 1). In doing so, Jull et al. [20] created a framework that advanced knowledge user engagement in health research because it provided an evidence-based, cohesive and concise approach to engaging knowledge users.

**Table 1:** Concepts of knowledge user engagement

Concept	Description of collaborative research process
Researcher: prepare, support	Initiate/support researcher capacity for power sharing, expertise, engagement (e.g., attending meetings with community groups)
Knowledge user: prepare, support	Initiate/support knowledge user/community organizational capacity for power sharing, expertise, engagement (e.g., provide training in research methods)
Relational process	Initiate and/or sustain a relational process between knowledge user and researcher to promote respect, reciprocity, trust and partnership synergy
Research agenda	Engage in a process to define study agenda (e.g., scope, priorities, objective(s))
Ethics: principles/values	Conduct knowledge user–researcher partnership work in an ethical way demonstrated by reflection on ethical concepts, concern with values and research conducted in ways reported as meaningful, respectful, inclusive of those in the research partnership
Research questions	Define research questions to identify what, specifically, the research project aims to achieve to justify the need to conduct the research
Resources	Develop funding applications/grant proposals to obtain resources to support knowledge user–researcher engagement
Ethics: policy/rules	Conduct knowledge user–research partnership work in an ethical way demonstrated by participation in an ethical application development (e.g., writing consent forms), review (e.g., research ethics board) and/or development and/or use of an ethical framework
Methodology	Decide on the research methodology or report process to justify the use of the proposed methodology
Methods	Decide upon research methods and a justification for the use of the proposed methods; selection of outcome measures
Collect data	Collect data. Includes tool development
Analysis	Decide on data analysis and interpretation (e.g., what form of analysis and how will be conducted)
Disseminate	Identify the appropriate audience to disseminate the research findings and tailor the message to the audience to create tangible products (e.g., publication of findings, community meetings)

“Concepts of knowledge user engagement” from Jull et al. [20] used under CC0 1.0 Universal Deed

Published studies about partnered rehabilitation research have contributed some understanding of partnerships. For instance, in a scoping review of partnered rehabilitation studies published up to 2013, Camden et al. found [17] that knowledge users most frequently engaged were individuals with disabilities and their families, rehabilitation clinicians and individuals representing community organizations or groups. They also found that the knowledge users were engaged in most steps of the research process, except for manuscript writing [17]. However, there is little published research on evaluation and effects of partnered rehabilitation research. Camden et al. [17] reported that only 32% of included studies evaluated the partnering process. Also, most effects identified by Camden et al. [17] were immediate or short-term, with few long-term effects of partnering reported. Additionally, in a 2019 study of partnered rehabilitation research projects from one provincial initiative in Quebec, Canada, Roberge-Dao et al. [28] found that outcomes of partnered rehabilitation research were also immediate or short-term, and specific to rehabilitation clinicians. To the best of our knowledge, no research has explored researchers’ and knowledge users’ perspectives on evaluating partnering in rehabilitation research. Evaluating the partnering process is important to accurately identify the short-, medium- and long-term effects of the partnering process, validate the assumptions of partnering, ensure integrity of partnering is upheld and provide insights to improve the partnering process [29–31]. The purpose of this study, situated within a Canadian context, was to explore researchers’ and knowledge users’ perspectives on evaluation of partnering in rehabilitation research, the effects of partnering on the research process and outcomes and how partnering contributed to the effects.

## Methods

### Design

We conducted a qualitative descriptive study, using virtual interviews. Qualitative description aims to accurately describe a phenomenon under study in concrete terms [32]. It is an appropriate design when the study focus is not well understood and can be used to provide practical answers to real-world problems [32–34]. The study was reported consistent with the Consolidated criteria for Reporting Qualitative Research (COREQ) checklist [35].

### Team positionality

B.J.T. identifies as a woman and was a PhD candidate at the time of this study, which was completed as part of her doctoral research. B.J.T. is a licensed physiotherapist and employed as an embedded researcher by a physiotherapy department in a publicly funded, tertiary care hospital in Canada. Her role is to promote the use of evidence to inform practice, policy and programming in the department. B.J.T. fulfils this role by partnering with employees on research and quality improvement projects that meet their needs. B.J.T. has observed how partnering on rehabilitation research facilitates evidence uptake in rehabilitation and its influence on practice, policy and program change. These experiences have informed her perceptions about partnered rehabilitation research, which influenced the interviews, analysis and interpretation of the results, particularly the tendency to view the results more positively than negatively. B.J.T. had prior qualitative research experience in conducting interviews and assisted with coding and theme development in thematic analysis.

The remainder of the research team are also women and faculty members at Canadian universities, who engage in partnered health or rehabilitation research and have extensive experience conducting qualitative research. Two team members are also rehabilitation professionals, i.e., occupational therapist (J.C.) and physiotherapist (L.C.L.). All authors are proponents of partnered health research and share an interest in critical reflection and evaluation of partnering in health and rehabilitation research in pursuit of enhancing the impact of health research in society.

### Ethical considerations

Ethics approval was obtained from the University of Manitoba Health Research Ethics Board (HS6260 (H2024:008)).

### Eligibility, sample and sample size and recruitment

Individuals were eligible if they were a researcher or knowledge user involved, or had been involved in partnered rehabilitation research, resided in Canada and understood and could communicate in English. We defined partnered rehabilitation research as a collaboration between researchers and knowledge users during the rehabilitation research process.

Our primary sampling approach was purposive, because it allowed us to intentionally choose eligible participants [36, 37]. By using purposive sampling, we believed we could involve a wide range of participants with diverse experiences and perspectives [36]. The following variables informed our purposive sampling: gender, geographical location, type of knowledge user (e.g., patient, clinician, decision maker), experience in partnered research and rehabilitation specialities. Secondly, we used snowball sampling which involved asking study participants and authors to share recruitment information within their networks to reach hard to recruit participants.

Our sample size was informed by data saturation [38], conceptual depth [39, 40] and existing research. A previous study by Roberge-Dao et al. [28] of partnered rehabilitation research achieved data sufficiency and generated themes following a focus group with six people and nine individual interviews, as such, we anticipated needing 15–19 participants. We initially conducted four interviews. After the initial group, B.J.T. reviewed new information generated after each interview in relation to the research and interview questions and consulted with K.M.S. Data collection was concluded when it was jointly agreed that minimal new insights were being shared.

We used multiple strategies for recruitment, and we used phased approach for recruitment to achieve a maximum variation sample. Firstly, B.J.T. asked the Canadian Physiotherapy Association, the Canadian Association of Occupational Therapists and Orthotists Prosthetics Canada to distribute a recruitment invitation to their members. This strategy reached approximately 26 000 people. Secondly, B.J.T. used the table of contents from Canadian rehabilitation speciality journals including The Canadian Journal of Occupational Therapy, Canadian Journal of Speech-Language Pathology and Audiology, Canadian Prosthetics & Orthotics Journal and Physiotherapy Canada between January 2022 and February 2024, to identify first authors of published rehabilitation research. Following this, B.J.T. emailed 108 authors an invitation to participate. Individuals who responded to the above recruitment strategies were emailed study information. Additionally, B.J.T. confirmed with participants where they lived, experiences in partnered research and rehabilitation and type of knowledge user. Recognizing most interested participants were predominantly researchers, we shared study information with our partnered rehabilitation research networks to reach more knowledge users. The recruitment strategy reached approximately 50 people.

### Consent and data collection

Eligible participants were contacted via email to schedule a virtual interview (MS Teams, Microsoft, Redmond, WA, United States of America). All participants were emailed the consent form which included a brief introduction about B.J.T. and explained the purpose of the study to review, sign and return to B.J.T. prior to interviews. Before each interview, B.J.T. reviewed the consent form and confirmed participants willingness to participate in the study. B.J.T. conducted and recorded interviews between January and April 2024. Participant identity was only known to B.J.T., who did not have a relationship with any participants.

All authors co-developed the semi-structured interview guide which included open-ended primary questions, follow up questions and associated probes informed by existing partnership literature, including the Jull and Wallerstein CBPR frameworks [20, 26–28]. For example, participants were asked to explain the effects of partnering on the research process, e.g., agenda setting, research questions, data collection, analysis, interpretation and dissemination, all concepts of knowledge user engagement identified by Jull et al. [20]. The Wallerstein CBPR model [26, 27], informed interview questions as well. For example, participants were encouraged to explain how they perceived partnering contributed to the effects they identified. We pilot tested the interview guide with three individuals with experience in partnered research for clarity and relevance. Based on pilot testing, we revised the interview guide to enhance clarity of the questions-based. Data from pilot testing were not included in analysis. See Additional file 1 for research objectives, corresponding interview questions and partnership literature that informed the questions. B.J.T. wrote field notes after each interview which documented contextual details about the interview, impressions, reflections and preliminary thoughts about data analysis. We asked all participants if they would like the opportunity to review the transcript to confirm their perspectives were accurately captured [36].

### Data analysis

Data analysis was led by B.J.T. and K.M.S. and was informed by Braun and Clarke’s [37, 41] guidance on reflexive thematic analysis, which is detailed in Additional file 2. Data analysis was completed inductively and deductively and used B.J.T.’s field notes to support analysis (described below). MS Teams (Microsoft, Redmond, WA, United States of America) was used for transcription and Microsoft Excel (Microsoft, Redmond, WA, United States of America) for data organization.

Data analysis was an iterative process that occurred simultaneously with data collection. After two interviews, B.J.T. de-identified and compared transcripts with video-recordings for accuracy. After this, B.J.T. immersed herself in the data by reading and rereading the transcriptions. B.J.T. repeated the process of interviewing, transcribing, reading and familiarizing herself with the data. After completing four interviews, B.J.T. developed a coding frame that combined inductive and deductive elements [36]. For example, B.J.T. inductively developed codes regarding the effects of partnering on the research process and outcomes directly from participant reflections because she was open to discovering new insights and understanding about effects of partnering without preconceived notions. However, B.J.T. deductively identified codes related to how partnering contributed to effects using concepts from the Wallerstein CBPR model [26, 27]. For instance, B.J.T. used codes related to context, relational, individual or structural dynamics that influence partnering and subsequently, identified effects.

The coding frame was refined collaboratively between B.J.T. and K.M.S. All transcripts were coded using the final coding frame to ensure accuracy. B.J.T. began the coding process by reading all the transcripts again. As she reread the transcripts, B.J.T. looked for interview data that was consistent with the codes [37, 42]. As B.J.T. coded the interview data, she began grouping codes with central concepts together, i.e., identified patterns in the codes [37, 42]. These central concepts became preliminary themes. As B.J.T. coded the data and started to generate preliminary themes, she began identifying quotes that could reflect the preliminary themes. Throughout coding, B.J.T. considered her assumptions of partnered rehabilitation research and how they informed her coding and preliminary theme development. Furthermore, B.J.T. drew on her experiences as an embedded researcher in a physiotherapy department who partners with employees to conduct research, to underpin her interpretation and impressions of the data. Lastly, as B.J.T. coded, she referred to her field notes to revisit her initial impressions, reflections and thoughts to support the patterns identified and preliminary themes developed.

As coding and theme development proceeded, all authors discussed the richness and meaning of the data gathered from the participants. In particular, we reflected on participant insights about partnering, which shaped our impressions. We compared and discussed participant data, which is consistent with participant triangulation, a strategy to strengthen the confidence in the research findings [36]. This work also ensured the preliminary themes were anchored to the participants’ perspectives and remained close to the dataset. Additionally, we discussed the relationships and connections between preliminary themes, whether there was sufficient data to support the preliminary themes, how the findings compared with existing literature and whether the preliminary themes answered the research questions, which is consistent with the process of determining data saturation and conceptual depth. Throughout discussions, we considered how our assumptions and experiences in partnered rehabilitation research affected interpretations about the data.

B.J.T. refined preliminary themes with K.M.S. and chose quotes for each theme. All authors discussed and queried the themes until we perceived they accurately reflected the data. The final themes and quotes were agreed upon by all authors and reflected the underlying meaning and patterns of the data. We took several measures to ensure trustworthiness of the interview data and analysis including employing member checks of the interview transcripts, keeping field notes, using participant triangulation and thick description and by staying close to the data.

## Results

Three organizations shared study information (the Canadian Physiotherapy Association, the Canadian Association of Occupational Therapists and Orthotists Prosthetics Canada), of which two people responded. Of the authors B.J.T. emailed, 21 people responded. Lastly, two people responded to emails sent by authors within their partnered rehabilitation networks. In total, 25 individuals expressed interest in the study. B.J.T. emailed 18 individuals with a range of rehabilitation experience i.e., as clinicians, researchers or knowledge users, to arrange an interview. One individual was ineligible because they had not been involved in partnered research. Another individual was not available. Three individuals did not respond to emails to arrange an interview. B.J.T. conducted 13 interviews between January and April 2024. We decided to stop data collection after 13 interviews, feeling we were acquiring minimal new information. As such, B.J.T. sent emails to the remaining seven individuals explaining that data collection was completed. Interviews lasted between 30–60 min.

### Characteristics of participants (Table 2)

**Table 2:** Characteristics of interview participants (*n* = 13)

Characteristics	*N*, (%)
Gender	*n* = 13
Female	12 (92)
Male	1 (8)
Race, ethnicity, or indigeneity	*n* = 13
White	12 (92)
Chinese	1 (8)
Province of residence	*n* = 13
British Columbia	4 (31)
Alberta	1 (8)
Manitoba	2 (15)
Ontario	5 (38)
Quebec	1 (8)
Type of partner	*n* = 13
Researcher	7 (54)
Knowledge user	6 (46)
Type of rehabilitation therapist (researcher only)	*n* = 6
Physiotherapist	3 (50)
Occupational therapist	2 (33)
Speech-language pathologist	1 (17)
Type of knowledge user	*n* = 6
Lived experience with a stroke	2 (33)
Lived experience with osteoporosis	1 (17)
Speech-language pathologist	1 (17)
Prosthetist	1 (17)
Leadership/policy development public healthcare system	1 (17)

Seven participants were researchers affiliated with an academic setting whose research included children, adults and older adults on a range of rehabilitation related topics, e.g., motor disabilities, homecare, falls rehabilitation, ageing and dementia, neurological conditions, use of power mobility, health professional education and reflective practice. Researchers had experience partnering with a variety of knowledge users, including those who had lived experience of a health condition, policy makers and rehabilitation clinicians. Researchers also had varying years of experience in partnered rehabilitation research; with most engaging in this approach for several years, and even decades, whereas one was just beginning. Six knowledge users participated, and most had been involved in research partnerships for five years. The knowledge users were either involved in a partnership at the time of their interview or had recently finished being involved in one.

### Perspectives on evaluation of partnering in rehabilitation research

None of the participants reported having the experience of formally evaluating the partnering process. When probed, both researchers and knowledge users recognized the value of conducting an evaluation. Some participants acknowledged evaluation may help to understand partnering in more depth and optimize how partners collaborated during the rehabilitation research process. One knowledge user (participant 11 (P11)) perceived that evaluation was beneficial because it could determine if knowledge users were involved in the partnership in a meaningful way and this was important because “… ensuring the patient perspective is included in research produces higher quality and better research.” Another knowledge user (P8) perceived that evaluation was beneficial for partnerships and could help everyone, “Get better at doing research together.”

Because of their typical role leading research, researchers were probed about why they did not evaluate the partnering process. Researchers perceived challenges to evaluation, in particular that evaluation was time-consuming process that may not be practical to carry out. For instance, one researcher (P4) questioned “…but is it realistic or practical? I don’t think so. It’s just endless, the list of partners, right.” Another researcher (P2) spoke about not wanting to overburden partners with another task as there were many expectations required of knowledge users to be involved in partnering. As this researcher indicated, “I think that I’m always worried about the burden of participation. So, I try not to add layers.” Furthermore, there was inconsistency in knowledge about the availability of existing tools for evaluation. For example, some researchers were unsure if tools existed, whereas other researchers were aware of tools to evaluate partnerships.

Even though the partnering process was not formally evaluated, researchers explained their processes. As one researcher (P4) explained, “I’m checking in with the partners that I’m engaged with, about how this [partnership] is going for them. Is there anything that’s kind of a barrier, is there anything that we can do to make things easier?” One knowledge user (P8) initiated their own “informal” evaluation of the partnering process and provided feedback to the research project team leaders. However, “the team leader was not open to the feedback.”

### Overarching theme and subthemes (Fig. 1)

**Fig. 1:**
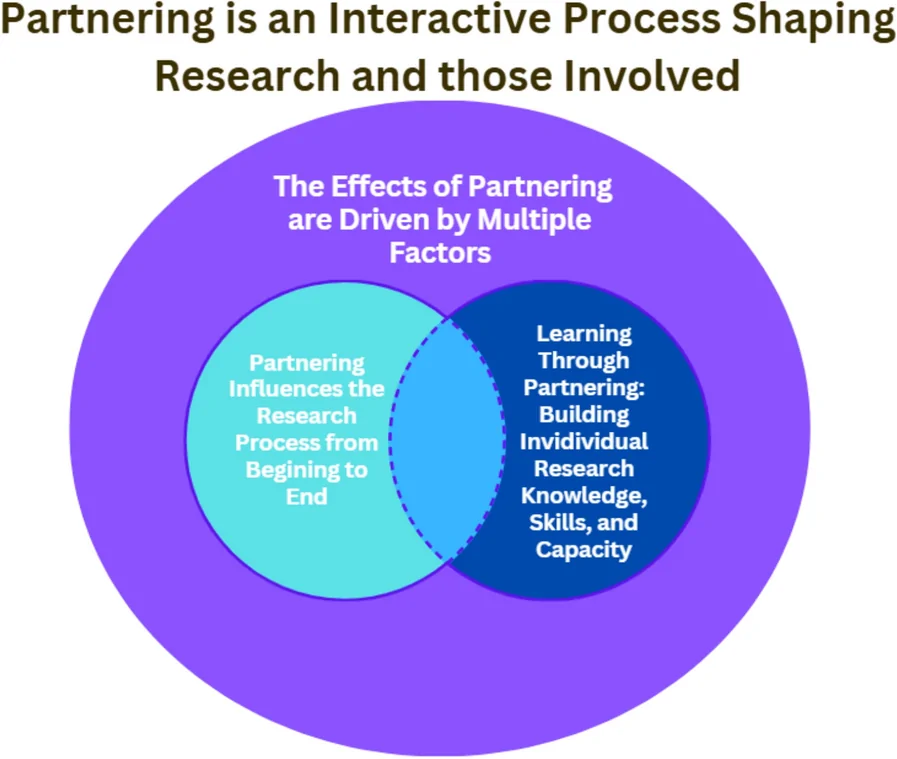
Themes and sub-themes about the effects of partnered rehabilitation research

Despite the lack of evaluation, all participants described effects of partnering on research process and outcomes, and how they believed partnering contributed to these effects. We generated one overarching theme, “Partnering is an Interactive Process Shaping Research and those Involved,” which captures participants’ perceptions that partnering produces effects across the research process, outcomes or those involved in partnered rehabilitation research. We created three subthemes: “Partnering Influences the Research Process from Beginning to End,” “Learning through Partnering: Building Individual Research Knowledge, Skills and Capacity” and “The Effects of Partnering are Driven by Multiple Factors.”

#### Partnering influences the research process from beginning to end

Participants identified effects that occurred throughout the life of a research project. Both researchers and knowledge users recognized that partnering facilitated the inclusion of a variety of perspectives in study planning and preparation, which was helpful because it forced partners to examine problems in different ways. Researchers identified that collaborating with knowledge users on developing research questions enhanced clinical relevance because they reflected real-world practice. Participants valued how different types of expertise strengthened the research design. As one researcher (P2) stated, “the study design is richer because you have broader and different types of expertise that are consulting on it.”

There were also perceived effects of partnering on recruitment, data collection and analysis/interpretation. Researchers perceived a key benefit of partnering was that data collection was optimized because knowledge users provided insight on whether methods were feasible. The critical role of knowledge users in data analysis and interpretation was recognized by both researchers and knowledge users in contributing to how findings could inform clinical practice. Participants also acknowledged practical contributions that facilitated the research process, such as the design of study advertisements. As one knowledge user (P11) explained, they advised the research team to, “…take off their ‘application writing skills’ and talk in a lay language” when designing advertisements to enhance recruitment.

Participants described how partnering in rehabilitation research influenced dissemination. Several knowledge users discussed their involvement in manuscript writing which they perceived as important because they could ensure findings were shared in an impactful way and the correct language was being used for the intended audience. Partnering also influenced dissemination. One researcher (P4) emphasized that during workshops or panel discussions knowledge users, “bring a perspective that researchers cannot. And so, and so it does help immensely with the effectiveness of the delivery or sharing or dissemination.”

Lastly, this sub-theme captures effects on research outcomes, some of which would be apparent after partnering concluded. For example, one knowledge user (P9) perceived that because their workplace was involved in partnering in rehabilitation research, it created, “… a legacy for our centre” and more employees became engaged in research. Furthermore, participants perceived the partnering process was the vehicle for establishing, strengthening and sustaining relationships amongst researchers and knowledge users, and was key to, “laying the foundation for partnerships that last over time.” (P3, researcher). Participants also spoke about how partnering lead to meaningful research findings, and effects of this, particularly on clinical practice. For example, one knowledge user (P13) was involved in a study that investigated prosthetic use by inpatients in a hospital setting. The study found that inpatients wore their prostheses very little when not participating in rehabilitation. This led to the development of recommendations to guide inpatients when to wear prostheses throughout the day, which were implemented by rehabilitation clinicians at the facility. As the knowledge user said about the findings, “… they were immediately used to change clinical practice.”

#### Learning through partnering: building individual research knowledge, skills and capacity

This sub-theme highlights that both researchers and knowledge users experienced individual effects that arose through partner interactions and active involvement in the research process. Participants described their learning as an individualized experience as a result of partnering. For instance, many researchers recognized partnering as an opportunity to learn from knowledge users and perceived partnering as a distinct skill that needed to be developed. One researcher (P7) believed this could ultimately, “… make me a better partnership partner [and] researcher.”

Both researchers and knowledge users recognized that partnering expanded their rehabilitation research capability, i.e., personal research knowledge and skills, such as learning about different research methodologies, different ways of thinking about research, developing research questions and how to answer those questions and writing manuscripts. As one knowledge user (P9) explained, partnering helped to teach them “…what are good questions to ask. And how to go about [answering] that.” Partnering was also an opportunity for knowledge users to learn to think critically about research. One knowledge user (P10), stated, “But I think also going through it ourselves was also helpful in understanding the limitations as well. You know both the good and the bad of research, right.”

Researchers valued how partnering increased their understanding of the public health care systems in their provinces and how services, such as rehabilitation, were organized and delivered. Furthermore, one researcher (P7) learned what types of outcome measures were being used clinically to assess speech and language pathology difficulties and this insight influenced their teaching at their university. Additionally, by partnering with rehabilitation clinicians in a hospital setting, the same researcher developed a better understanding of research and quality improvement that was being conducted within their local health care system, the types of internal funding resources that were available to clinicians and the support that managers provide to front-line clinicians to participate in rehabilitation research.

#### The effects of partnering are driven by multiple factors

This sub-theme captures researchers’ and knowledge users’ perceptions on the contextual, structural, relational and individual factors which shaped how the partnering process unfolded. The factors either enhanced or hindered partnering, thus influencing how partnering contributed to perceived effects.

##### Contextual factors

Contextual factors refer to socio-economic, historical elements, university and community elements that influence the partnering process [27]. Knowledge users highlighted how support from departmental or organizational leaders contributed to positive effects of partnering. One knowledge user who was employed in a private (i.e., for profit) healthcare setting shared how the leadership at their organization appreciated, prioritized and supported evidence-based practice and research. As this knowledge user (P9) stated, “…I think it was easier [to partner] because of having that support and not having to fight for it, right?” This organization wanted to be a centre of excellence and believed that partnering with an academic institution to work together on rehabilitation research was one way to achieve this goal.

However, researchers perceived several contextual factors beyond their control that influenced the partnering process. For instance, researchers spoke about the challenges with obtaining funding to support the partnering process. One researcher described how they often self-funded partnering activities, at least until they received grant money. Furthermore, researchers shared it was often a challenge convincing ethics boards that compensation for knowledge users was reflective of the expertise they brought to the partnership, and not coercion. Another researcher (P2) perceived that funding agencies required knowledge users to, “jump through hoops to be on a grant” and relayed that knowledge users had a lot of paperwork and training to complete to partner. Researchers also perceived that knowledge users didn’t always understand components of the research process and the time it takes to conduct research well. However, one researcher (P3) also acknowledged some ignorance of clinical contexts and their influence on the partnering process, “Because I think you know the realities of clinical practice and the time constraints and the competing demands. Researchers don’t always understand those things because they’re not in that environment.”

##### Structural factors

Structural factors refer to formal partnering agreements that helped to govern the partnering process [27]. One researcher (P4) explained that they had a point of contact person for the partnering process who was, “An individual [that] coordinates and makes sure that everyone is doing what they should be doing.” Researchers also used terms of reference materials, research agreements or charters to facilitate the research and partnering process. One researcher described their research agreement as a guide that would keep all the partners on the right track while they were collaborating on a research project. Another researcher (P2) explained they utilized a charter which was “… a document that says, like, this is what our objective is. This is how often we’re going to meet. This is the authorship rules. These are your options for communicating.”

##### Relational factors

Relational factors, that is, how the partners behaved and interacted together, influenced partnering and subsequently, the effects of partnering. Participants described many relational factors that influenced the effects of the partnerships including trust, reliability and accountability, role definition and clarity, showing an interest in partners, respect, recognition, acknowledgement and acknowledging that knowledge users were valued.

“I think also it was really nice knowing that as an academic, she respected what we saw as a research question, right. So that was the other thing is that there was this, it wasn’t lesser than or you know just because it was coming from a clinical place.” P9 (knowledge user).

Participants emphasized how power imbalances or differentials influenced partnering. Many participants spoke about how all partners should be on equal footing and there must be a level playing field for everyone during partnering. Researchers recognized their responsibilities in this area and spoke about specific actions to reduce power differentials: “I only use my first name. I don’t wear a lab coat.” (P5). However, some knowledge users highlighted relational factors that negatively influenced partnering. For instance, they spoke about not being given the opportunity to provide feedback on research applications and not being listened to. One knowledge user (P8) was invited to partner on a study with a researcher and had several years of lived experience with a health condition but described their experience as feeling “like a token; not being heard, listened too, and understood. I felt like a voice on the margin.”

##### Individual factors

Both researchers and knowledge users stated that individual factors such as interest, curiosity and enthusiasm, being open-minded, adaptable and motivated and working together on a research problem also contributed to positive effects of partnering. Researcher humility, that is, acknowledging the limits of one’s knowledge, was another individual factor that participants felt contributed to positive effects of partnering. As one researcher (P3) stated, “You have to acknowledge that what you see as a researcher is one tiny slice and you really have to be reliant on others to guide you.” Lastly, as one knowledge user with lived experience of a health condition (P8) noted, there was usually only one type of partner represented, “that is, the middle-class, white women.”

## Discussion

The aims of this qualitative descriptive study situated in a Canadian context were to explore evaluation, perceived effects of partnering during rehabilitation research on the research process and research outcomes, and how partnering contributed to the effects. Our study produced findings that can be used to guide future research within this area. The lack of formal evaluation of partnered rehabilitation research is a meaningful finding as it consistent with Camden et al. [17] who found only one third of studies included in their scoping review evaluated the partnering process in rehabilitation research. This suggests that the evaluation of partnering remains stagnant, which is a concern as evaluation can help to identify short-, medium, or long-term effects. However, we did not limit our inclusion criteria to those who evaluated partnering. As such, we recognize there could be value in exploring the experiences and perspectives of researchers and knowledge users who have evaluated the partnering process – this may provide insights into how they evaluate – barriers and facilitators to evaluating, and the effects of partnering.

Despite the lack of formal evaluation, researchers explained they “informally checked-in” with knowledge users about their satisfaction with the partnerships. Camden et al. [17] also reported informal check-ins were a common strategy to touch-base with knowledge users. Camden et al. [17] referred to this approach as “debriefing,” and it only occurred after the partnership ended. While informal check-ins may be helpful to determine if changes need to be made to the partnering process, using tools or instruments to evaluate the partnering process may accurately capture effects without relying solely on researchers’ or knowledge users’ perceptions. The use of high-quality standardized tools or instruments to evaluate partnerships may be particularly helpful to identify medium- or long-term effects of the partnering process on health outcomes or the healthcare system, evidence which is currently lacking. Such instruments exist, and future studies could investigate the acceptability, appropriateness and feasibility of partnership evaluation tools identified by Mrklas et al. [43] for use in partnered rehabilitation research.

Even though participants did not evaluate the partnering process, they believed evaluation was valuable. This is a meaningful finding because it demonstrates a positive attitude towards evaluation, and this positive attitude may influence future behaviour [44]. However, in our study, acknowledging the value of evaluation did not translate into evaluating the partnering process. When questioned why evaluation was not completed, researchers perceived challenges with evaluation. Understanding existing barriers is helpful as these results may guide interventions which could facilitate evaluation of the partnering process. Future analyses using established behavioural models such as the Capability, Opportunity, Motivation (COM-B) model [45], could be used to examine barriers and facilitators to the evaluation of partnering. These findings could inform interventions which support evaluation of partnering in rehabilitation research.

Despite hypotheses that partnering can enhance effects on the uptake of evidence thus minimizing the evidence–practice gap, participants did not provide many insights into this. Only one knowledge user described a concrete example of how the partnering process enhanced the use of evidence in clinical practice and changed practice recommendations. We were surprised that more participants didn’t provide examples of uptake of evidence in practice. It is concerning that partnered research presents itself as a solution to the gap between evidence and practice considering the limited evidence to substantiate this assumption. However, only two knowledge users in our study were rehabilitation clinicians and we hypothesize if we had more clinicians, we may have heard more examples of how partnering enhanced evidence uptake. Furthermore, we speculate that minimizing the evidence–practice gap through changes in rehabilitation practice, i.e., behaviour, could take prolonged periods of time. In fact, these long-term effects may not be realized until the partnership is over. The use of formal evaluation to assess the partnering process may be suited to capture these long-term effects after the partnering process has concluded.

Findings provided insights into how aspects of partnering, such as contextual (e.g., management support), structural (e.g., point of contact person, charters), individual (e.g., interest, curiosity) and relational dynamics (e.g., power differentials), contributed to effects. Our findings align with existing research [17, 25, 46]. Kothari et al. [46] conducted a realist review that examined how integrated knowledge translation (IKT) works. IKT is a partnering approach to research, and Kothari et al. [46] found that infrastructure (e.g., leadership, resources), role clarity and power sharing are integral conditions necessary for effective partnerships. Additionally, Camden et al. [17] found that power sharing, a relational factor, was essential for partnered research. Power sharing established a common ground for partners and helped to promote an environment which allowed for negotiation of study agendas, resolved conflicts and supported meaningful engagement, teamwork and collaboration [17]. Rycroft-Malone et al. [47] highlight that partnered approaches to research should be equity-driven and ensure they include processes for power sharing. However, imbalances in power-sharing within partnered health research continue and create hierarchies between researchers and knowledge users [48]. These hierarchies perpetuate unconscious biases, and patient partners report that they perceive that their lived experiences are given less credit and respect by researchers, which is consistent with what one knowledge user expressed in our study [48]. Consequently, patient partners report feeling less important, not as smart as other team members or “lesser” than other partners [48]. Rycroft-Malone et al. [47] acknowledge relinquishing power in partnered research can be a challenge but argue researchers must adapt to an equitable way of conducting research with knowledge users. To do so creates an experience where differences in knowledge, perspectives and viewpoints are equally embraced and have the capacity to contribute valuable insights into health research [47].

Our findings also confirm understanding about the individual effects of partnering. Studies about partnered health research have previously reported that partnering increased knowledge, capacity and skills for research, confidence, self-efficacy, enhanced health behaviours, feeling valued and building trust [25, 49, 50]. Additionally, findings from partnered health research have identified and described principles and strategies that can be used to plan and conduct partnered research, thus potentially enhancing the optimization of these partnerships, leading to positive effects [25]. Many of the participants from our interviews identified factors that were consistent with these principles and strategies including respect, role clarity and definition, the use of terms of reference or charters and engaging with knowledge users in various phases of the research process [50].

## Limitations

Our sample primarily identified as women and White. We theorize this likely represents a larger systemic issue regarding lack of representation of Black, Indigenous and People of Colour (BIPOC) individuals in academia, specifically rehabilitation research and rehabilitation educational training programs. Additionally, the under-representation of BIPOC knowledge users may reflect their personal experiences with the healthcare system. BIPOC people are more likely to experience racism in healthcare [51, 52] and this may influence their interest in being involved in partnered rehabilitation research. We also note that individuals had to understand English to be eligible and this language restriction may have dissuaded some individuals from participating. Lastly, involvement in the study was voluntary and may reflect the perspectives of individuals with a vested interest in partnered rehabilitation research.

## Conclusions

The absence of evaluation is problematic as the full complement of effects related to partnering may not be identified yet. The challenges study participants associated with evaluating the partnering process may drive a lack of engagement with formal evaluation activities. Notwithstanding the benefits perceived by participants, evaluation solutions that balance rigour with relevance warrant continued consideration to ensure all voices are represented. Future work may consider who is best suited to lead evaluation of the partnering process and how best to support evaluation. Given the continued weighting of perceived effects on individual and research process outcomes, ongoing effort is needed to support evidence uptake for patient outcomes.

## Supplementary information

Additional file 1.

Additional file 2.

## Data Availability

The data that support the findings of this study are available from the corresponding author, BJT, upon reasonable request.
