# Supplementary material for: A qualitative descriptive study to explore evaluation and effects of partnered rehabilitation research in Canada
Source: Health Res Policy Syst. 2026 Jun 26;24:78. doi: 10.1186/s12961-026-01505-1 (PMC13579909; doi:10.1186/s12961-026-01505-1)
Supplement: Supplementary file 1 [file 12961_2026_1505_MOESM1_ESM.docx]

Additional file 1: Interview questions

|  | **Research objective** | |
| --- | --- | --- |
|  | - Explore the effects of partnered research on the research process. | |
|  | **Interview question (s)** | **Reference** |
|  | How do you think researchers and knowledge users working together affected the research process? These can be positive or negative effects. Take your time and provide as much detail as you would like.  *Probe:* When I say ‘research process’ I am referring to setting the project’s priorities, choosing research questions, development of the study design and methods, choosing study outcomes, developing ethics documents, recruiting participants, collecting, and analyzing data, and interpreting and sharing results with academic and non-academic populations, and sustainability of interventions. What is your experience like as a [researcher or knowledge user] collaborating with [researcher or knowledge user] on any of these aspects of the research process and what were the effects?  *Probe:* To clarify, I am not asking about the effects of the research project itself. For example, I am not asking about whether the research project improved patient related outcomes. Rather, I am specifically asking about the collaboration between the researchers and knowledge users and whether it affected the research process. For example, what effect (or consequence) was there by involving the knowledge users in decision making related to the research process?  *Probe:* In your view, what were some of the good and/or bad things that happened when **you** partnered with [researchers or knowledge users] in the research process?  Do you think anything about the research process would have been different if knowledge users were not on the research team?  I noticed you didn’t say [insert research process not mentioned]. Did you consider involving knowledge users [or being involved] in this part of the research?  Do you think it would have made a difference if knowledge users were involved in this phase of the research process? | Jull et al^18^ |
| **Research objective** | | |
| - Explore the effects of partnered research on research outcomes. | | |
| **Interview question (s)** | | **References** |
| In your view (or experience), how did researchers and knowledge users working together affect the results or research outcomes? These can be positive or negative effects. Take your time and provide as much detail as you would like.  *Probe:* When I say ‘research outcomes’ I am referring to health system policy changes, sustainability of partnerships, knowledge user empowerment, shared power relations, cultural revitalization, knowledge users’ knowledge about the research process, knowledge users’ research skills, researchers’ capacity for partnered research, reduction of health disparities, production of useful research findings for knowledge users, and evidence-informed clinical decision making by knowledge users.  *Probe:* To clarify, I am not asking about the effects of the research project itself. Rather, I am specifically asking about the collaboration, partnering, and working together between the researchers and knowledge users and whether it affected research outcomes. A research outcome may be research or knowledge user capacity for partnered research.  *Probe:* What were some of the good and/or bad things that were the result of the partnership on the results or research outcomes?  Were there any other effects on the results or research outcomes that we have not discussed in the previous question that you experienced? | | Jull et al^18^ |

| **Research objective** | |
| --- | --- |
| - Explore how effects of the partnership were evaluated. | |
| **Interview question (s)** | **References** |
| Can you tell me if the effects of the partnership you mentioned in the previous questions were formally evaluated or measured?  *Probe:* When I say “formally” evaluated or measured, I am referring to using specific methods, like a survey, interview, or focus group, to measure the effects.  [If answer “yes” to question 6] Can you tell me how the partnership was evaluated or measured?  *Probe:* For instance, did you have to fill out a questionnaire or survey? Did you talk about them in an interview or focus group?  I noticed you said, ‘questionnaire or survey’. Do you remember if you developed those together, as a research team. Or did you use a questionnaire or survey that was already developed?  So, you developed the questionnaires and surveys as a team. How did this happen? For instance, did you have meetings to talk about what should go into the questionnaire or survey? How did you decide what went into the questionnaire or survey?  [If answer “yes” to question 6] Can you explain, or do you recall why the approaches you mentioned were used to evaluate the partnership? It is okay if you are unsure or do not know.  *Probe:* For instance, did you decide on those approaches as a group. Or did someone tell you that was how they would be evaluated.  *Probe:* Were the approaches chosen because of the research groups’ familiarity with them? Or for convenience?  [If answer “yes” to question 6] Do you think there is value in evaluating the partnership? Why or why not?  [If answer “no” to question 6] To clarify, the partnership (s) that you were involved in was not formally evaluated. Why do you think that was?  Do you think there is any value in evaluating the partnership? Why or why not? | Roberge-Dao et al^26^ |

| **Research objective** | |
| --- | --- |
| - Explore how partnered rehabilitation research affects the research process and outcomes. | |
| **Interview question (s)** | **References** |
| How do you think the collaboration between researchers and knowledge users led to effects on the research process?  *Probe:* For instance, you mentioned the partnership resulted in [interviewer insert answers from question 1]. In what ways did the researchers and knowledge users collaboration contribute to these effects on the research process?  What is it about [answer from question 11] that contributed to effects of the partnership?  How do you think the collaboration between researchers and knowledge users during rehabilitation research led to effects on the research outcomes?    *Probe:* For instance, you mentioned the partnership resulted in [interviewer insert answers from question 4]. In what ways did the researchers and knowledge users collaboration contribute to these effects on the research process?  What is it about [answer from question 12] that contributed to effects of the partnership? | Jull et al^18^  Wallerstein et al^24^  Belone et al^25^  Wallerstein et al^24^  Belone et al^25^  Jull et al^18^  Wallerstein et al^24^  Belone et al^25^ |
