# Supplementary material for: A qualitative descriptive study to explore evaluation and effects of partnered rehabilitation research in Canada
Source: Health Res Policy Syst. 2026 Jun 26;24:78. doi: 10.1186/s12961-026-01505-1 (PMC13579909; doi:10.1186/s12961-026-01505-1)
Supplement: Supplementary file 2 [file 12961_2026_1505_MOESM2_ESM.docx]

Additional file 2: Reflexive thematic analysis as per Braun and Clarke^35,38^

| **Stage of thematic analysis** | **Description** |
| --- | --- |
| Transcription | Recorded interviews transcribed verbatim. |
| Reading and familiarization | Immersing self in the dataset by reading, & re-reading data, to become very familiar with them; Gaining loose impressions or concepts/ ideas of the data. |
| Coding | Identifying aspects of data that relate to research questions; Coding can be complete or selective; Codes are a word or phrase that capture the essence of a bit of data that may be useful; Codes are the building blocks of analysis; Data can be coded in multiple ways; Codes can be data derived or researcher derived; Coding can be completed many ways. |
| Generating themes | Deeper analysis to identify patterns in the codes; Looking for ideas that recur across the dataset; Patterns to capture the different elements that are most meaningful to answer the research question; A theme is broader than a code and is the central organizing concept; A theme contains lots of different ideas related to centralizing concept; Codes combine to form themes; Review codes looking for similarity; A theme can be distinct but still fit together with other themes; May have overarching themes and/or sub-themes; Themes are provisional. |
| Reviewing themes | Process to ensure that the themes fit well with the coded data and the research question; Involves re-reading codes, collated data, and the whole data set; Aim to ensure the themes capture the meaning of the data in relation to the research question |
| Defining and naming themes | Process of defining the focus, scope, purpose, and/ or boundaries of the themes; typically, a few short sentences. |
| Writing and finalizing analysis | Process of writing; Analysis develops; Narrative that is written which tells the story of the theme about the content and meaning of the data; Select vivid and compelling extracts from the data to illustrate facets of the theme; Can use an illustrative approach for a thick and rich description of the data; Present themes logically; Situate final analysis in current literature. |
